# Bis-indole derivatives with antitumor activity turn out to be specific ligands of human telomeric G-quadruplex

**DOI:** 10.3389/fchem.2014.00054

**Published:** 2014-07-24

**Authors:** Jussara Amato, Nunzia Iaccarino, Bruno Pagano, Rita Morigi, Alessandra Locatelli, Alberto Leoni, Mirella Rambaldi, Pasquale Zizza, Annamaria Biroccio, Ettore Novellino, Antonio Randazzo

**Affiliations:** ^1^Department of Pharmacy, University of Naples “Federico II”Naples, Italy; ^2^Department of Pharmacy and Biotechnology (FaBiT), University of BolognaBologna, Italy; ^3^Experimental Chemotherapy Laboratory, Regina Elena National Cancer InstituteRome, Italy

**Keywords:** G-quadruplex, anticancer activity, telomeric damage, thermal stabilization, phenanthroline derivatives, pyridine derivatives

## Abstract

Bis-indolinone derivatives having either 2,6-disubstituted pyridine core (**1a** and **1b**) or 1,10-disubstituted phenanthroline core (**2a** and **2b**), already known to have antitumor activity, have been tested as potential G-quadruplex binders. Compounds **2a** and **2b** are able to selectively stabilize G-quadruplex over duplex DNA, and also to discriminate among different G-quadruplex structures, having a particular affinity for the parallel form of the human telomeric G-quadruplex. Both compounds are also able to induce telomeric DNA damage that may explain the activity of these compounds.

## Introduction

G-quadruplexes (G4) are four-stranded nucleic acid structures that spontaneously form within G-rich sequences of DNA and RNA in the presence of cations (Bochman et al., 2012). The recent unambiguous evidence of G4 formation in living cells has increased the enthusiasm and has propelled numerous investigations in this field (Biffi et al., 2013). Several experiments have located G4-forming sequences in different critical positions of the human genome, mainly at the telomeric and gene promoter level (Bochman et al., 2012). In particular, the telomeric regions at the chromosome ends play a critical role in the regulation of cellular proliferation. They are made up by 2–20 kb of double-stranded TTAGGG repeats and feature a 3′ single-stranded overhang of 50–500 nucleotides (Wright et al., 1997). Parallel to normal cells proliferation, telomeres get gradually shorter, triggering irreversible cellular growth arrest (senescence) (Harley et al., 1990; Price, 1999). A telomere maintenance mechanism is provided by the six-membered protein complex called shelterin and by telomerase. The latter adds copies of the repeated motif to the end of the single-stranded overhang. This enzyme is transcriptionally repressed in most differentiated human somatic cells while being overexpressed in about 85% of cancer cells (Kim, 1997; Shay and Wright, 2005). In the remaining 15% of human tumors, telomere lengthening is obtained by a different mechanism known as alternative lengthening of telomere (ALT) (Fajkus et al., 2005). In both cases, telomeres are maintained to a stable length with consequent senescence circumvention and cellular immortalization. It has been shown that the 3′ G-rich single-stranded overhang of the human telomeric DNA can adopt G4 structures and that the formation of the G-quadruplexes inhibits telomerase activity *in vitro* (Zahler et al., 1991). Furthermore, it has also been demonstrated that molecules that stabilize telomeric G-quadruplexes increase the inhibition of the telomerase (Sun et al., 1997) and lead to telomeric protein uncapping, which, in turn, leads to the onset of DNA damage responses and cellular apoptosis. This has opened a new drug intervention field in anticancer therapy. Several different classes of ligands that target G4 DNA have been developed (Granzhan et al., 2010; Monchaud et al., 2010; Ohnmacht and Neidle, 2014). A number of these have been identified by our research group and most of them were discovered in order to target the grooves of the G4 structures (Cosconati et al., 2009, 2010, 2012; Pagano et al., 2010; Petraccone et al., 2011; Di Leva et al., 2013). On the other hand, several other research groups have developed molecules characterized by an extended planar aromatic scaffold, which is generally able to stack on the external G-tetrads of the G4. Compounds having a central pyridine (like, for example, pyridostatin and 360A) (Granotier et al., 2005; Rodriguez et al., 2008) or 1,10-phenanthroline (like, for example, PhenDC3, and PhenDC6) moieties (Dhamodharan et al., 2012) belong to this latter group.

**Figure 1:**
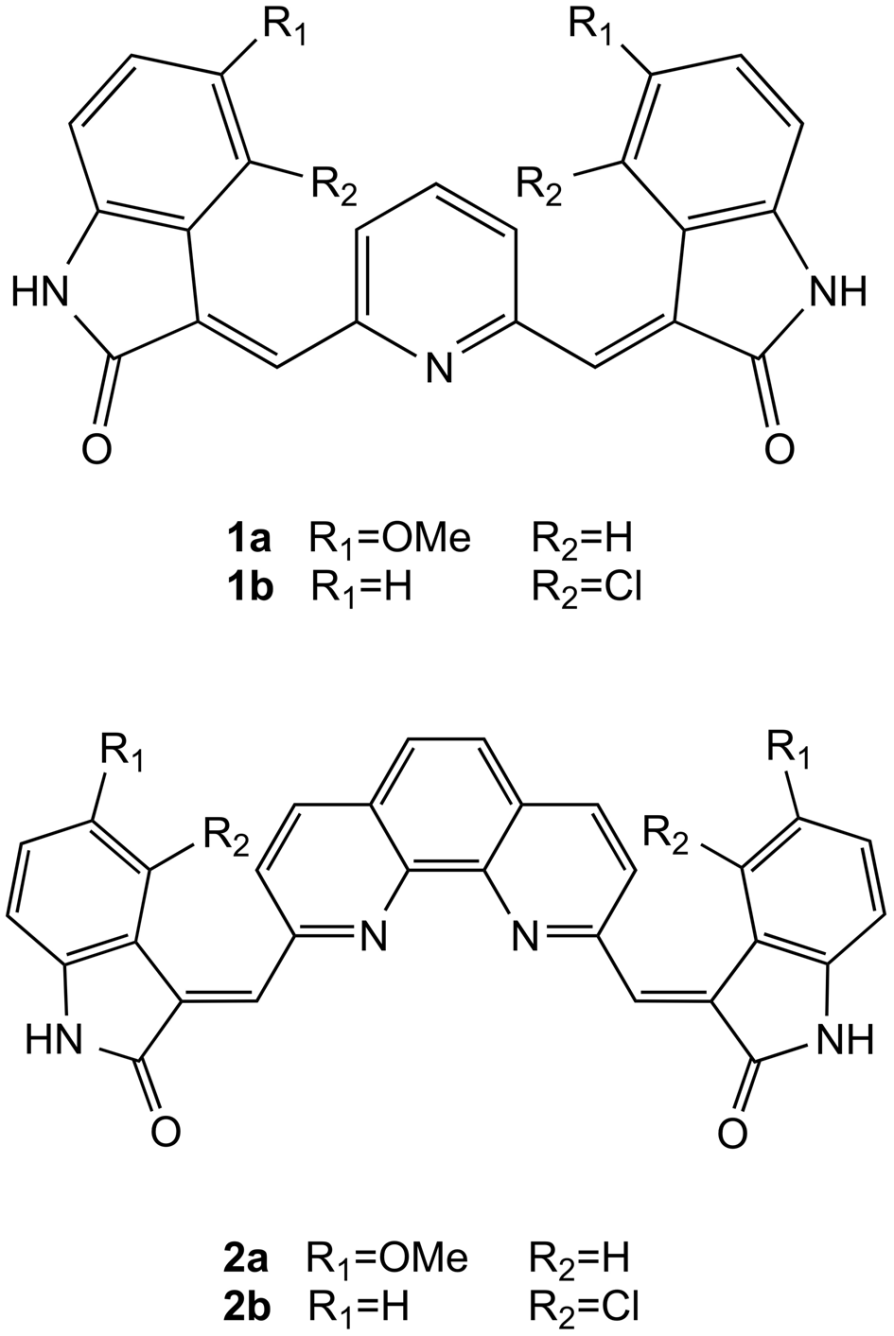
**Chemical structures**. Chemical structures of compounds **1a,b** and **2a,b**.

Recently, some of us have synthesized and successfully tested very similar molecules as antitumor agents: the bis-indolinone derivatives with the 2,6-disubstituted pyridine core (**1a** and **1b**) as well as the same derivatives with the 1,10-disubstituted phenanthroline core (**2a** and **2b**) (Figure 1) (Andreani et al., 2008, 2010). Interestingly, the structural similarities of these compounds with the mentioned G4 binders inspired us a further investigation in order to evaluate the G4 binding properties of **1a**,**b** and **2a**,**b**, and possibly to propose a potential mode of action of these derivatives capable to explain their antitumor activity. In particular, in this paper we report the results of the binding studies of compounds **1a,b** and **2a,b** with different G-quadruplex topologies, along with their capability to induce telomeric damage.

## Materials and methods

### Oligonucleotides

All synthetic oligonucleotides have been purchased by Biomers (Germany), purified employing standard HPLC protocols and checked for their integrity by MALDI mass spectrometry. In particular, the following DNA sequences have been used for the experiments: two different truncations of human telomeric DNA sequence, namely 5′-TAGGGTTAGGGTTAGGGTTAGGG-3′ (**tel23**) and 5′-TTAGGGTTAGGGTTAGGGTTAGGGTT-3′ (**tel26)**; two sequences from the promoter region of the *c-kit* oncogene, namely 5′-AGGGAGGGCGCTGGGAGGAGGG-3′ (**ckit1**) and 5′-CGGGCGGGCGCGAGGGAGGGG-3′ (**ckit2**); the self-complementary duplex-forming Dickerson dodecamer 5′-CGCGAATTCGCG-3′ (**ds12**).

### Preparation of the sample

G-quadruplexes were prepared in the appropriate buffer (10 mM Li_3_PO_4_, 50 mM KCl, pH 7.0 for **ckit2**; 10 mM Li_3_PO_4_, 100 mM KCl, pH 7.0 for all the other oligonucleotides) at 10 μM single strand concentration, unless otherwise stated. The solutions have been annealed by heating at 90°C for 5 min, and gradually cooling to room temperature overnight. The concentration of all oligonucleotides was measured at 260 nm by UV measurement at 90°C using the appropriate molar extinction coefficients. Parallel arrangement of **tel23** oligonucleotide was obtained as reported in the literature (Renciuk et al., 2009), by annealing of 10 mM single strand oligonucleotide in 10 mM Li_3_PO_4_, 100 mM KCl, pH 7.0. After annealing, the concentrated DNA solution was kept at 4°C for 24 h before dilution. After dilution (necessary for spectroscopic measurements), the concentration of the sample was refined by measuring absorption at 260 nm, using a molar extinction coefficient appropriate for these conditions. To verify that the dilution did not alter the species in solution, CD spectral changes with time were checked, without any appreciable change observed over the period of time required to complete the experiments.

### Circular dichroism (CD) spectroscopy

CD spectra and CD melting curves of oligonucleotides were recorded on a Jasco J-715 spectropolarimeter equipped with a Jasco PTC-423S Peltier temperature controller. CD spectra were recorded in the wavelength range 230–360 nm at 20°C, with a scan rate of 100 nm/min, a response time of 1 s and a bandwidth of 1 nm. All the spectra were averaged over 3 scans. Buffer baseline was subtracted from each spectrum. The DNA concentration was 10 μM (as single strand) and ligand stock solution was 1.5 mM in DMSO. DNA/ligand mixtures were obtained by adding 4 molar equiv. of ligands (40 μ M). CD melting were performed in the temperature range 20–100°C, at the heating rate of 1°C/min by following changes of the CD signal at the wavelengths of maximum variations upon oligonucleotide folding. The melting temperatures were determined from fit of melting curves using two state transition model implemented in Origin 8.0 program. Each melting experiment was performed at least three times.

### Gel electrophoresis

Native gel electrophoresis analysis was carried out on 15% polyacrylamide gel at 5°C, which was run in 1×TB (pH 7.5) buffer supplemented with 50 mM KCl. An oligonucleotide concentration of 50 μM was used for each sample. Various amounts (2–4 equiv) of ligands **1a**,**b** and **2a**,**b** were incubated with DNA at 25°C for 1 h before loading. Prior to loading the mixtures onto the gel, 1 μL of glycerol solution (60% v/v) was added. The total volume loaded in each well was 10 μL.

### Molecular docking

The crystal structure of the 23-mer human telomeric G-quadruplex DNA 5′-TAGGGTTAGGGTTAGGGTTAGGG-3′ bound to a tetra-substituted naphthalene diimide ligand (PDB code 3CDM) was used as the target for docking studies (Parkinson et al., 2008). The ligand was removed from the structure to leave empty binding sites. The parallel topology of the structure results in accessible external 5' and 3' planar G-tetrad surfaces, defined as the grid box, being the potential binding sites for the ligands. The size of the box was constrained to 15 × 15 × 15 Å in the x, y, and z dimensions. After optimizing the ligands and assigning partial atomic charges, docking calculations were performed with AutoDock4.0 program using Lamarckian genetic algorithm (Morris et al., 2009). Grid maps were generated for each atom type in the ligand using AutoGrid. An active site box was created with a grid spacing of 0.375 Å. The maximum number of energy evaluations was set to 1.0 × 10^6^, the maximum number of genetic algorithm operations was set to 2.7 × 10^4^, the number of individuals in population was set to 150, the rate of mutation and crossover were set to 0.02 and 0.8, respectively. When searching the conformational and orientational spaces of a ligand with rotatable bonds having full flexibility, the structure of the G-quadruplex was kept rigid. 20 independent dockings were carried out to evaluated different ligand poses.

### Cells and culture conditions

BJ fibroblasts expressing hTERT plus SV40 early region (BJ-HELT) were obtained as previously reported (Salvati et al., 2010). The cell line was grown in Dulbecco Modified Eagle Medium (D-MEM, Invitrogen Carlsbad, CA, USA) supplemented with 10% fetal calf serum, 2 mM L-glutamin and antibiotics.

### Immunofluorescence

Immunofluorescence (IF) was performed as previously described (Salvati et al., 2007). Briefly, cells were fixed in 2% formaldehyde and permeabilized in PBS plus 0.25% Triton X-100 for 5 min at room temperature. For immunolabeling, cells were incubated with primary antibody for 2 h at room temperature, washed twice in PBS and finally incubated with the secondary antibodies for 1 h. The following antibodies were used: rabbit policlonal anti-TRF1 antibody (Abcam Ltd.; Cambridge UK); mouse monoclonal anti-γH2AX antibody (Upstate, Lake Placid, NY); TRITC-conjugated Goat anti-Rabbit, FITC-conjugated Goat anti Mouse (Jackson Immunoresearch, Suffolk, UK). Nuclei were immunostained with DAPI. Fluorescence signals were recorded by using a Leica DMIRE2 microscope equipped with a Leica DFC 350FX camera and elaborated by Leica FW4000 deconvolution software (Leica, Solms, Germany). For quantitative analysis of γH2AX positivity, 200 cells on triplicate slices were scored. For TIF analysis, a single plane was analyzed and 30 γH2AX-positive cells were scored. Cells with at least 4 co-localizations (γH2AX /TRF1) were considered as TIF-positive.

### Statistical analysis

The biological experiments have been repeated three times and the obtained results are presented as means ± standard deviation (SD). Significant changes were assessed by using Student's *t*-test for unpaired data, and *P*-values < 0.05 (*) were considered significant.

## Results and discussion

### Target selection

In order to investigate the G-quadruplex binding properties of compounds **1a**,**b** and **2a**,**b** (Figure 1), a number of G-quadruplex forming sequences were selected for this investigation. In particular, we focused our attention on both telomeric and non-telomeric sequences able to form G-quadruplexes and having different folding topologies.

**Figure 2:**
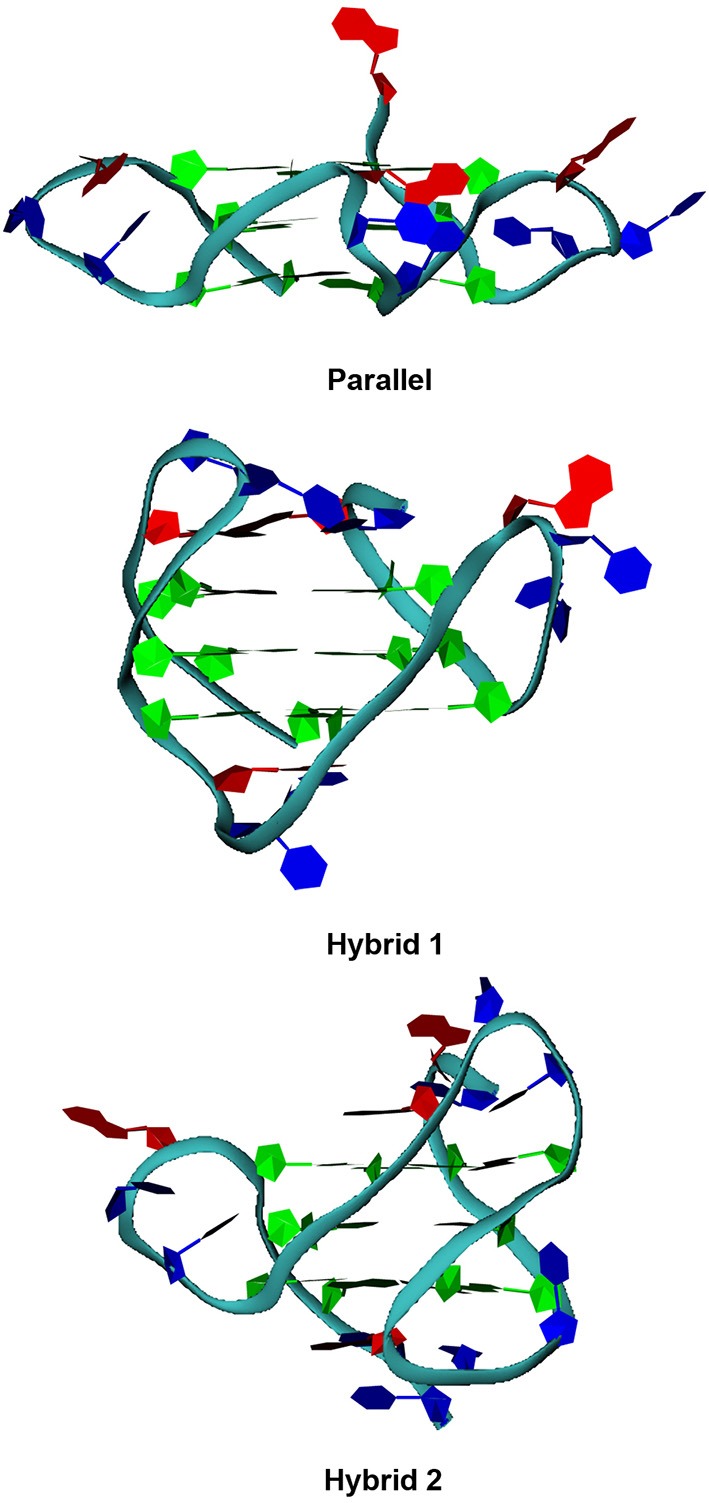
**G-quadruplex folding topologies**. Pictorial representations of the parallel, hybrid 1 and 2 folding topologies of human telomeric G-quadruplexes.

As far as telomeric DNA is concerned, it is well known that, in the presence of K^+^, it can fold into a variety of G-quadruplex topologies depending on experimental conditions and length of the sequences (Dai et al., 2008). Since this can have important implications in drug discovery, we selected telomeric DNA truncations and experimental conditions such as to have three different folding topologies, which possess most of the structural features of numerous folding topologies of telomeric DNA. Thus, we considered two different truncations of human telomeric DNA sequence, namely **tel23** and **tel26** (Material and Methods). Vorlickova and co-workers have demonstrated that a high DNA concentration promotes the G4 parallel folding of human telomeric sequence (Renciuk et al., 2009) and that, although intermolecular species may be formed at high concentrations, the majority of oligonucleotides form intramolecular G4 structures. Thus, we prepared a couple of sample of **tel23** that were structured at “low concentration” (10 μM) and “high concentration” (10 mM) conditions, respectively. Particularly, at “low concentration” conditions, **tel23** is expected to form the so-called *hybrid 1* G4 structure (Figure 2) (hereafter referred to as **tel23-h**), whereas, at “high concentration” conditions, the **tel23** is expected to fold into a G4 parallel structure (hereafter referred to as **tel23-p**). On the other hand, the sequence **tel26** at 10 μM is expected to fold into the *hybrid 2* G4 structure (Figure 2). In order to verify that these sequences actually adopt the expected folding, CD experiments were performed. Indeed, CD is a well-established technique for determining the presence and the overall topologies of G4 structures (Masiero et al., 2010; Karsisiotis et al., 2011; Randazzo et al., 2013), although it should be noted that the interpretation of CD spectra requires spectra of well characterized G4 structures for comparison. The **tel26** sequence showed a CD spectrum having two positive bands at 290 and 268 nm, and a weak negative band at around 240 nm (Figure S1, Supplementary Material). These data are consistent with a *hybrid 2* G4 folding topology. Very similar CD spectrum was obtained for **tel23-h**, indicating also in this case an antiparallel G4 folding topology (namely *hybrid 1*) (Figure S1, Supplementary Material). **Tel23-p** actually adopts a parallel conformation, having positive band around 265 nm and a negative band around 240 nm in the CD spectrum (Figure S1, Supplementary Material).

As mentioned above, we also took into consideration two non-telomeric sequences (see Materials and Methods) and both of them were analyzed by CD spectroscopy. Particularly, we prepared the promoter DNA sequence **ckit1**, which exists as a parallel structure (Phan et al., 2007), having a characteristic positive band at 262 nm and a negative band at 240 nm in the CD spectrum (Figure S1, Supplementary Material). The other target G-quadruplex considered is that formed by the **ckit2** sequence, that exists in a dimeric parallel-stranded conformation (Kuryavyi et al., 2010) as indicated by a major positive band at 262 nm and a negative band at 240 nm.

Finally, in order to evaluate the selectivity of the ligands for the G4 over the duplex DNA, we used as target also the Dickerson duplex-forming dodecamer (**ds12**).

### Binding analysis

Circular dichroism (CD) studies were performed to explore the potential of the new ligands to alter the native folding topology of the investigated G-quadruplexes, inducing a particular conformation. Thus, the bis-indolinone derivatives with the 2,6-disubstituted pyridine core (**1a**,**b**), as well as the derivatives with the 1,10-disubstituted phenanthroline core (**2a**,**b**) were synthesized as previously described (Andreani et al., 2008, 2010). Upon addition of an excess of ligands (4 equivalents relative to the DNA), no relevant variations of DNA chiroptical signal were observed for all the analyzed structures (Figure S1, Supplementary Material), thus suggesting an overall conservation of the G4 structures as well as of their architectures. Analogously, the interaction between the ligands and the Dickerson duplex-forming dodecamer (**ds12**) was also investigated by CD spectroscopy to evaluate their effect on the reference DNA duplex. The CD spectra of **ds12** in the absence and in presence of ligands in solution were almost superimposable (Figure S1, Supplementary Material), thus suggesting that the investigated compounds do not alter the duplex structure.

**Figure 3:**
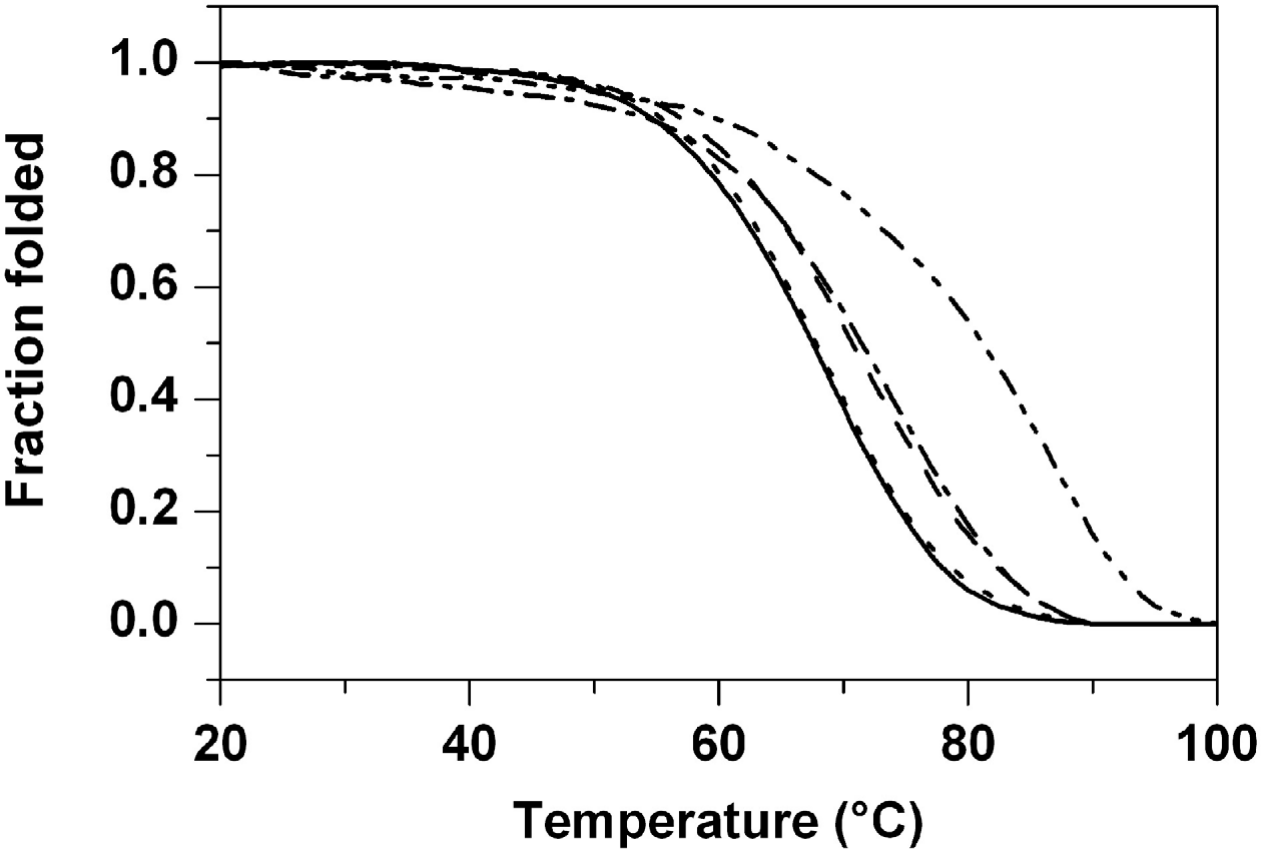
**CD melting experiments**. Normalized CD melting curves of **tel23-p** in the absence (solid) and in presence of 4 molar equiv of ligands **1a** (dash), **1b** (dot), **2a** (dash dot), and **2b** (dash dot dot). *T_m_* values are listed in Table 1.

Then, the DNA-stabilizing properties of the compounds were evaluated by measuring the ligand-induced change in the melting temperature (Δ*T_m_*) of the various G4-forming sequences as well as of the duplex-forming sequence in CD melting experiments (Giancola and Pagano, 2013). All the thermal denaturations were monitored at the wavelengths of maximum CD intensity. In particular, the melting profiles of the parallel G4 structures were recorded at 264 nm (**tel23-p**) and 262 nm (**ckit1**, **ckit2**), while the thermal denaturations of the hybrid-type G4s were monitored at 289 nm (**tel23-h**) and 290 nm (**tel26**) (Figure S2, Supplementary Material). Instead, CD melting curves of **ds12** duplex were recorded at 280 nm. Ligands **1a** and **1b** did not increase significantly the stability of any G4 DNAs as well as of duplex (Table 1). On the other hand, ligands **2a** and **2b** enhanced the stability of the parallel telomeric G4 **tel23-p** by 5.5 and 15.5°C, respectively (Figure 3 and Table 1). Very interestingly, the same ligands showed to induce only a slight increase (up to 3.0°C) of thermal stability of all the other investigated G4s and none for the duplex. These results highlight the fact that ligands **2a** and **2b** not only selectively stabilize G4 over duplex DNA, but also discriminate among different G-quadruplex structures.

**Table 1:** **Melting temperatures**.

**Ligands**	***T_m_*(°C)**					
	**ckit1**	**ckit2**	**tel23-p**	**tel23-h**	**tel26**	**ds12**
No ligand	70.5 (±0.2)	72.5 (±0.2)	68.5 (±0.3)	64.5 (±0.2)	66.5 (±0.2)	64.0 (±0.3)
**1a**	71.0 (±0.3)	75.5 (±0.2)	71.5 (±0.2)	64.5 (±0.3)	66.5 (±0.3)	64.5 (±0.2)
**1b**	71.0 (±0.2)	74.0 (±0.3)	68.5 (±0.3)	64.5 (±0.2)	66.5 (±0.3)	62.5 (±0.2)
**2a**	71.0 (±0.2)	75.0 (±0.2)	74.0 (±0.3)	65.0 (±0.2)	66.5 (±0.3)	64.5 (±0.3)
**2b**	72.0 (±0.3)	75.0 (±0.3)	84.0 (±0.3)	64.5 (±0.3)	66.5 (±0.2)	64.5 (±0.2)

*Melting temperatures of G-quadruplex and duplex DNAs with and without ligands measured by CD melting experiments*.

**Figure 4:**
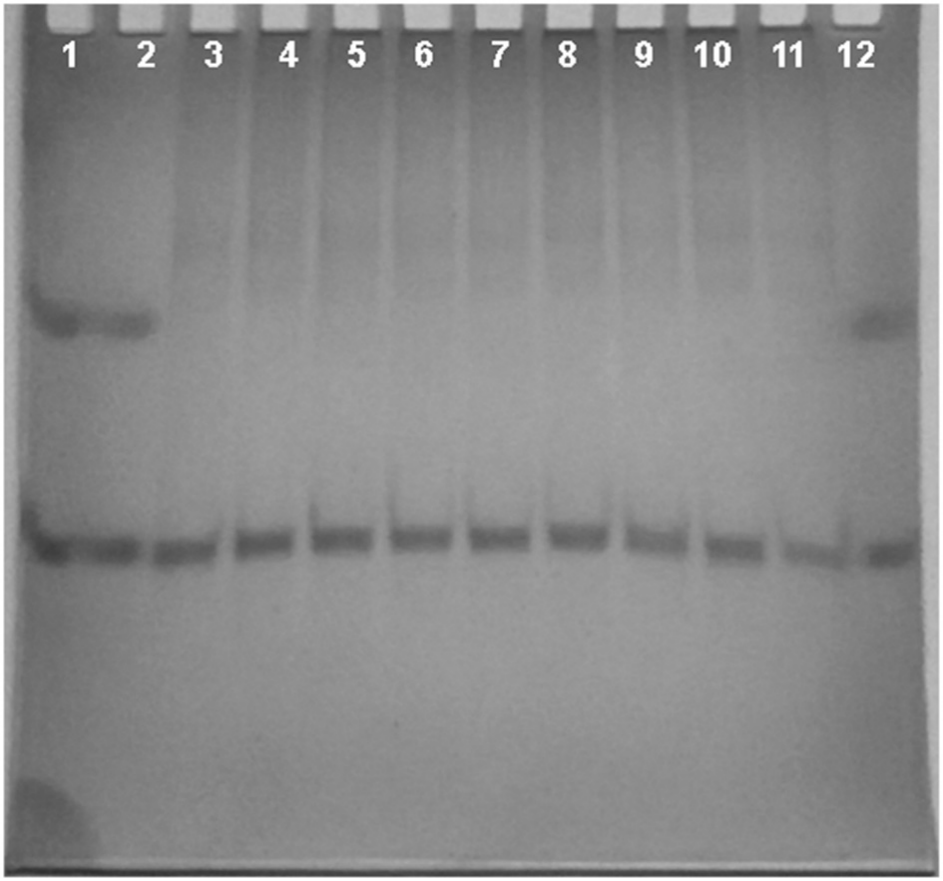
**Nondenaturing PAGE**. Nondenaturing PAGE of human telomeric DNA **tel23-p** (50 μM) with increasing equivalents of ligands (2 and 4 eq.) at 5°C. Lanes 1 and 2: bromophenol blue; lane 3: **tel23-p** alone; lane 4: [**tel23-p**+**1a**] 1:2 mixture; lane 5: [**tel23-p**+**1a**] 1:4 mixture; lane 6: [**tel23-p**+**1b**] 1:2 mixture; lane 7: [**tel23-p**+**1b**] 1:4 mixture; lane 8: [**tel23-p**+**2a**] 1:2 mixture; lane 9: [**tel23-p**+**2a**] 1:4 mixture; lane 10: [**tel23-p**+**2b**] 1:2 mixture; lane 11: [**tel23-p**+**2b**] 1:4 mixture; lane 12: bromophenol blue.

Nondenaturing gel electrophoresis experiments were performed on **tel23-p**, that is the G-quadruplex more stabilized by the ligands. In particular, the experiments were performed before and after the addition of the ligands, to confirm the presence of the intramolecular G4 structure as major conformation in solution. As shown in Figure 4, **tel23-p** moves essentially as single band in the gel, thus suggesting the absence of high-order structures. Moreover, the addiction of ligands did not have any pronounced effect on the G4 mobility. This clearly indicates that (i) all investigated ligands do not induce DNA dimerization/oligomerization, (ii) in agreement with CD results these ligands do not promote any G4 conformational change.

### Biological and molecular activity

**Figure 5:**
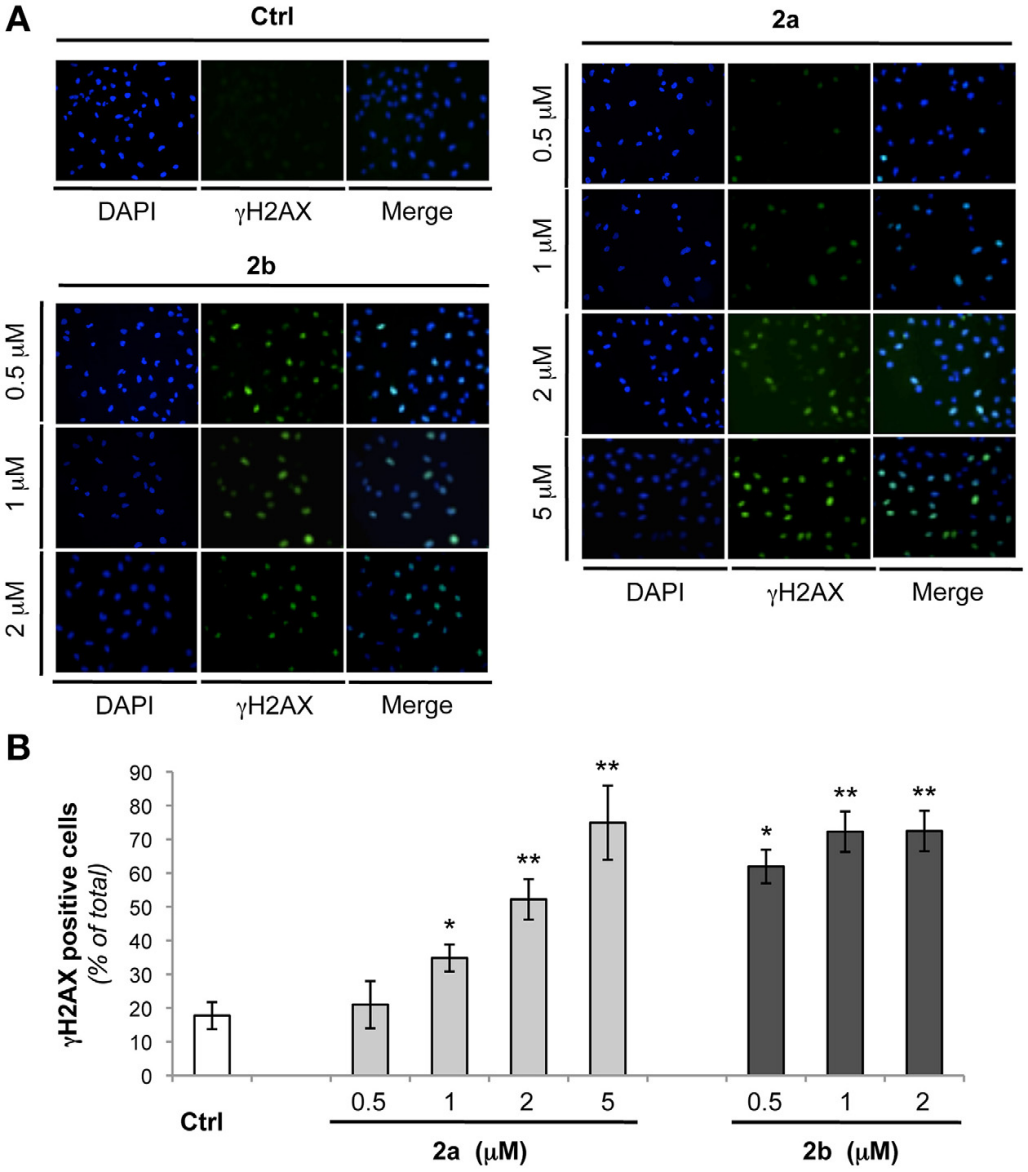
**Analysis of DNA damage response by bis-indole derivatives 2a and 2b**. Transformed BJ-EHLT fibroblasts were grown for 24 h in absence (−) or in presence of the indicated concentrations of compound **2a** or **2b**. DNA damage response was evaluated by immunofluorescence (IF) analysis by using an anti-γH2AX antibody (green) and DAPI (blue) was used to mark nuclei. **(A)** Representative images of IF analysis. Images were acquired by using a Leica Deconvolution microscope (magnification 20×). **(B)** Quantification of γH2AX-positive BJ-EHLT fibroblasto from **(A)**. Histograms show the mean values ± *SD* of at least three independent experiments. *p*-values were calculated using the student *t*-test (^*^p < 0.05; ^**^p < 0.005).

The two molecules that were able to significantly increase the thermal stability of the telomeric G4 were further investigated from biological point of view. In particular, we evaluated if the mechanism through which the two bis-indole derivatives **2a** and **2b** exert their antitumor activity is due to their ability to bind the G4 DNA structures. Thus, human transformed fibroblasts (BJ-EHLT) were exposed for 24 h to different concentrations of the two compounds and activation of DNA damage response (DDR) was evaluated by immunofluorescence. As shown in Figure 5, both ligands, even if at different extents, induced the phosphorylation of H2AX, a hallmark of DDR at almost all the drug doses tested (Thiriet and Hayes, 2005). Specifically, treatment with compound **2a** produced a dose-dependent effect with an induction of γH2AX-positive cells starting from 1 μM (about 30%) and reaching about 70% of positive cells at 5 μM concentration (Figure 5). Interestingly, exposure of BJ-EHLT to 0.5 μM of **2b** was already sufficient to induce a potent phosphorylation of H2AX (more than 50% of positive cells), percentage that does not further enhanced with the increase of the dosage (Figure 5). Altogether, these results suggest that the chemical substituents introduced in the tested molecules can determine a different affinity of the two ligands for the target.

**Figure 6:**
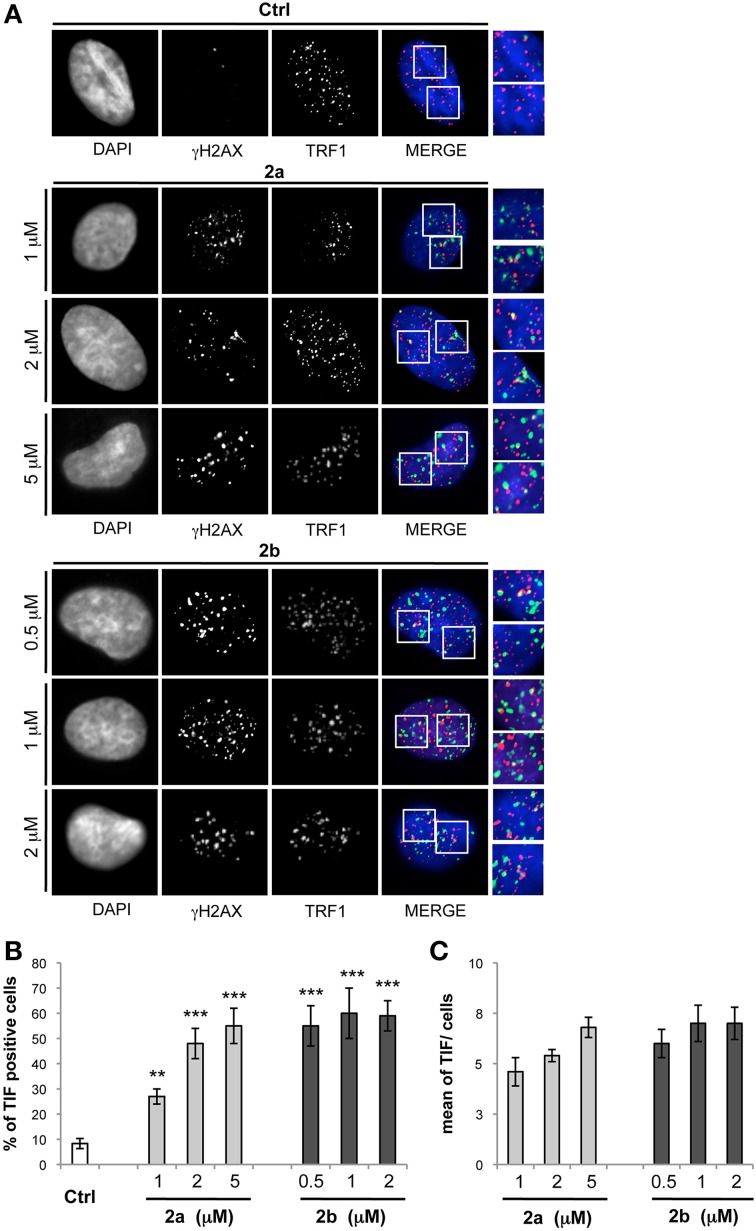
**Analysis of telomere damage by bis-indole derivatives 2a and 2b**. BJ-EHLT fibroblasts were grown in absence (−) or in presence of the bis-indole derivatives **2a** and **2b** at the reported concentration. Upon 24 h, each sample was processed for IF analysis by using antibodies against γH2AX (green) and TRF1 (red) to mark DNA damage and telomeres, respectively. DAPI staining was used to mark nuclei. **(A)** Representative IF images acquired by using a Leica Deconvolution microscope (magnification 63×). Enlarged views of TIFs are reported on the right of each picture. **(B,C)** Quantification of TIF-positive cells **(B)** and mean of TIFs per nucleus **(C)** from IF experiments reported in **(A)**. Data are means ± *SD* of three independent experiments. *p*-values were calculated using the student *t*-test (^**^p < 0.005; ^***^p < 0.001).

To evaluate whether γH2AX was phosphorylated in response to dysfunctional telomeres, the most effective drug concentrations of both compounds were tested by double immunofluorescence (IF). The analysis performed by deconvolution microscopy revealed that both compounds induced γH2AX foci that colocalized with TRF1, an effective marker for telomeres, generating the so-called telomere-dysfunction induced foci (TIFs) (Takai et al., 2003) (Figure 6), clearly indicating that the tested compounds caused telomere localized damage. Consistent with these data, results from quantitative analysis revealed that both **2a** and **2b** significantly increased the percentage of cells with more than four γH2AX/TRF1 colocalizations (Pearson's correlation coefficient ≥0.45), with a mean of about 6 TIFs per nucleus (Figure 6).

Notably, while at the lowest doses of **2a**, the majority of γH2AX spots colocalized with TRF1, at the highest concentrations an important fraction of the damage was not localized at the telomeres. On the contrary, compounds induced both telomeric and not telomeric DNA damage even at low drug doses (0.5 μM concentration). This is in line with recent data in which it has been demonstrated that G-quadruplex structures have been found in the promoters of several genes involved in cancer processes (e.g., c-myc, bcl-2, VEGF) an now visualized in extra-telomeric regions of human cells (Biffi et al., 2013).

### Molecular docking

**Figure 7:**
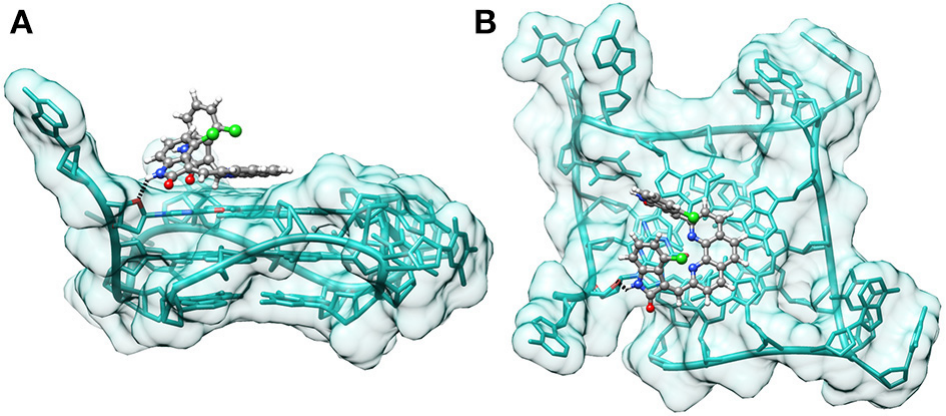
**Binding pose of 2b on tel23-p**. Side **(A)** and top-view **(B)** of the binding pose of **2b** on **tel23-p** obtained by docking calculations. Hydrogen bond between the indolinone moiety and the O4' atom of a deoxyribose ring is depicted with a dashed black line.

In order to understand the mode of binding and the fit of the best ligands (**2a,b**) within the **tel23-p** G-quadruplex structure (the most stabilized G4) we performed molecular docking calculations. Among computational methods, molecular docking is one of the most important techniques, and it has been widely used to predict or to give insight into the interaction between small ligands and biological macromolecules (such as proteins and nucleic acids). As more and more G4 structures have been determined (Neidle, 2009), a number of novel ligands have been discovered using this technique (Cosconati et al., 2009; Alcaro et al., 2012; Pagano et al., 2012). We docked the ligands to an X-ray crystal structure of the parallel 23-mer human telomeric G4 (PDB ID 3CDM) using AutoDock (Morris et al., 2009). For each ligand, the most favorable complex was selected from the docked structures on the basis of the calculated binding energies. As expected, in both cases, the predicted most favorable binding mode was one where the phenanthroline core is parallel to the plane of the terminal G-tetrad, making extensive π –π stacking interactions. Noteworthy, in the case of **2b**, we observed that the NH group of one of the indolinone systems is hydrogen bonded to the O4' atom of a deoxyribose ring (Figure 7). On the other hand, **2a** does not seem capable of forming this additional interaction, probably because of the different spatial arrangement of the indolinone systems, which seem to be involved in an intramolecular lone pair-π stacking interaction, conferring rigidity to the molecule (Figure S3, Supplementary Material). This could justify the higher ability of **2b** to increase the thermal stability of the telomeric G4.

## Conclusions

We have synthesized two bis-indolinone derivatives with the 2,6-disubstituted pyridine core (**1a** and **1b**) and two other derivatives having a 1,10-disubstituted phenanthroline core (**2a** and **2b**). These compounds have shown interesting antitumor activity, even if their mode of action is unknown. Interestingly, the structural similarities of these molecules with well-known G-quadruplex binders like for example pyridostatin (Granotier et al., 2005; Rodriguez et al., 2008) or PhenDC3 and PhenDC6 (Dhamodharan et al., 2012) suggested us that also these molecules could actually bind G-quadruplexes and this interaction maybe responsible of their antitumor activity. Compounds **2a** and **2b** actually are the only two compounds able to selectively stabilize G4 over duplex DNA and also to discriminate among different G-quadruplex structures, having a particular affinity for the parallel human telomeric G-quadruplex **tel23-p**. Docking calculations have indicated potential binding modes for those compounds, providing possible explanations of the different affinities and activities, and therefore laying the basis for the development of new ligands. All together these results represent the proof of concept that **2a** and **2b** interact and stabilize the G4 structures both *in vitro* and *in cellulo*, and therefore that they could be considered as the lead compounds for developing new anticancer drugs.

### Conflict of interest statement

The authors declare that the research was conducted in the absence of any commercial or financial relationships that could be construed as a potential conflict of interest.
