# Supplementary material for: Bis-indole derivatives with antitumor activity turn out to be specific ligands of human telomeric G-quadruplex
Source: Front Chem. 2014 Jul 24;2:54. doi: 10.3389/fchem.2014.00054 (PMC4109613; doi:10.3389/fchem.2014.00054)
Supplement: Supplementary file 1 [file DataSheet1.DOCX]

1. **Supplementary Material**

**
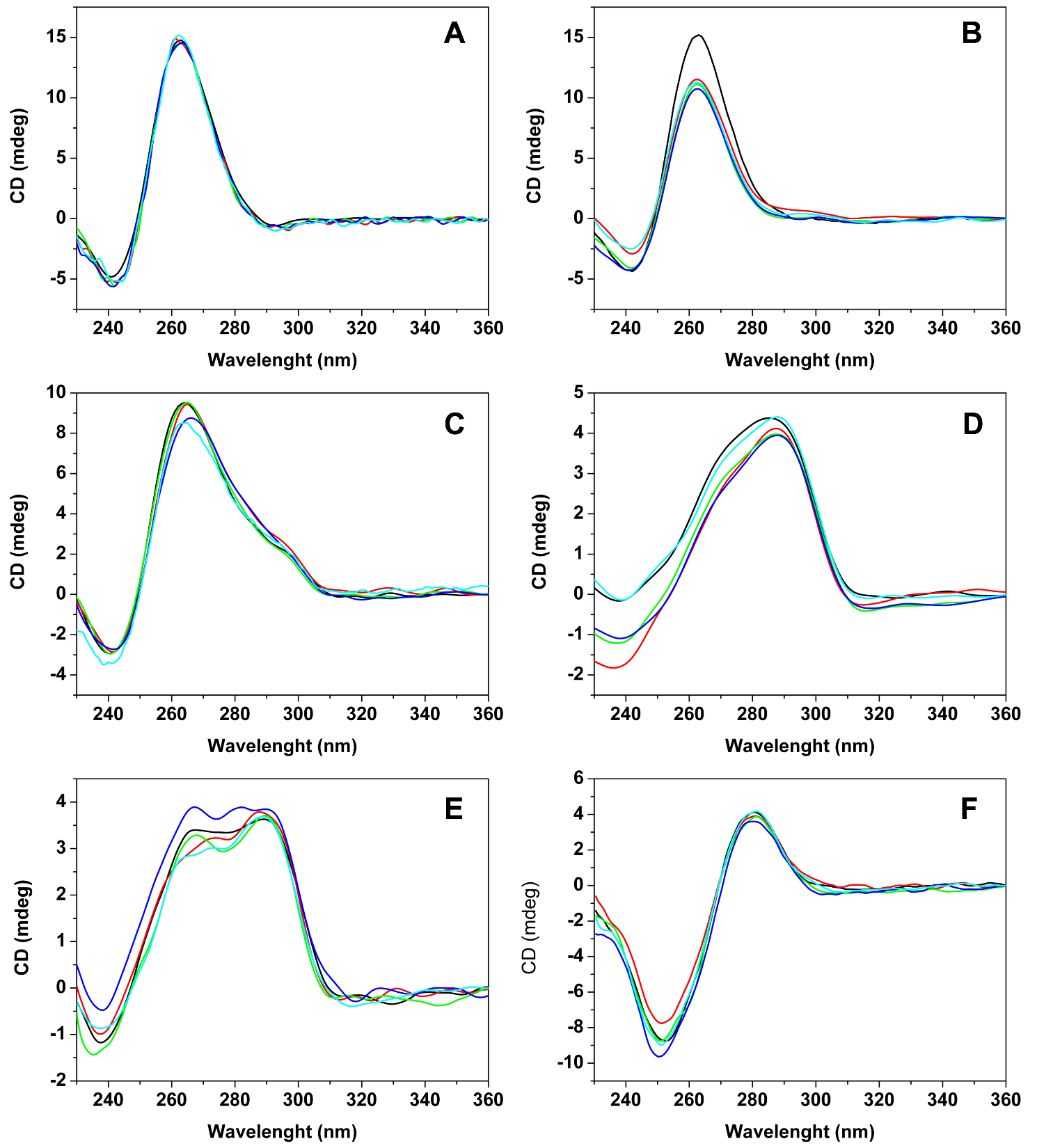
**

**Supplementary Figure 1. CD spectra.** CD spectra of **ckit1** (A), **ckit2** (B), **tel23-p** (C), **tel23-h** (D), **tel26** (E), and **ds12** (F) in the absence (black line) and in presence of 4 molar equiv of ligands **1a** (red line), **1b** (green line), **2a** (blue line), and **2b** (cyan line).


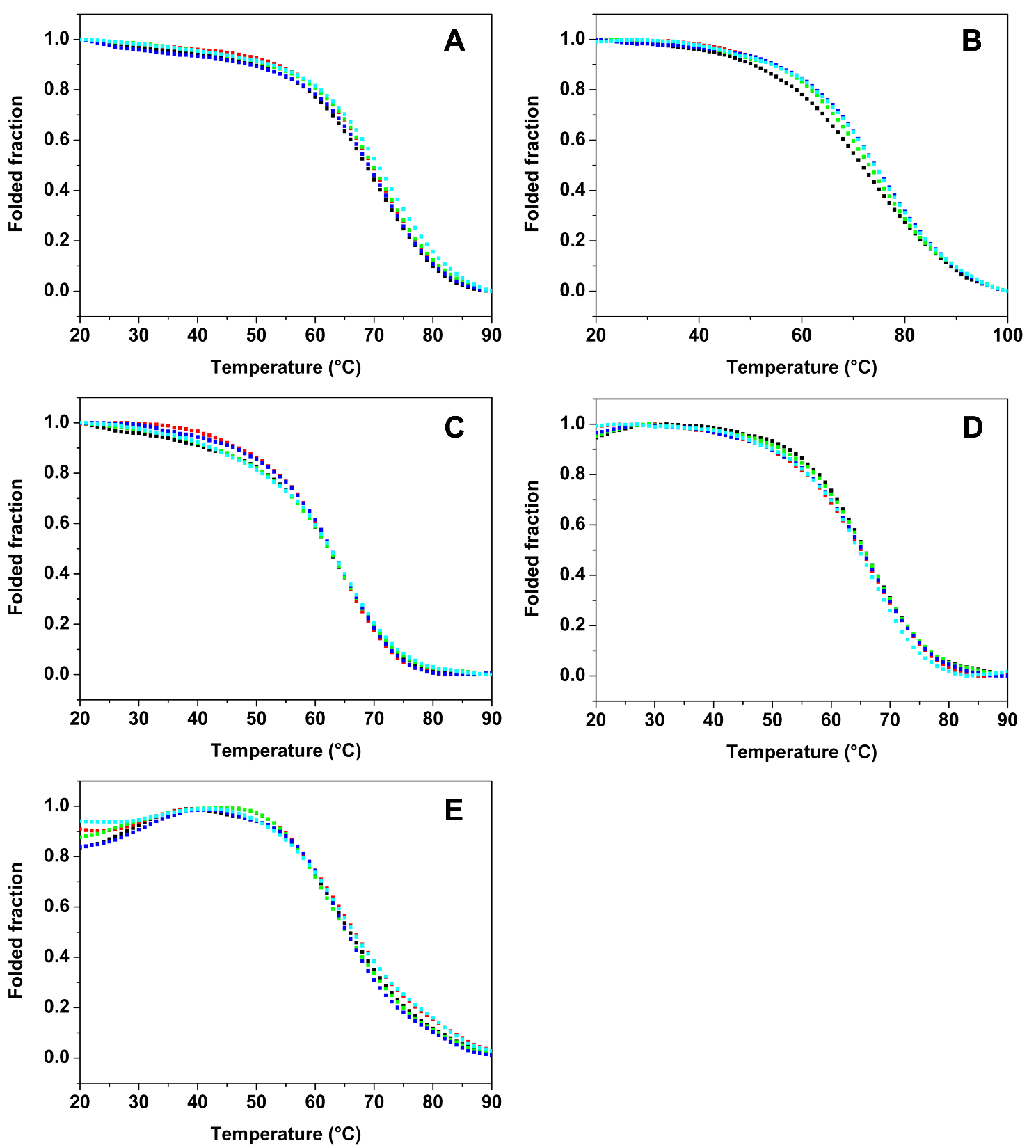


Supplementary Figure 2. CD melting. Normalized CD melting curves of ckit1 (A), ckit2 (B), tel23-h (C), tel26 (D), and ds12 (E) in the absence (black squares) and in presence of 4 molar equiv of ligands 1a (red squares), 1b (green squares), 2a (blue squares), and 2b (cyan squares).


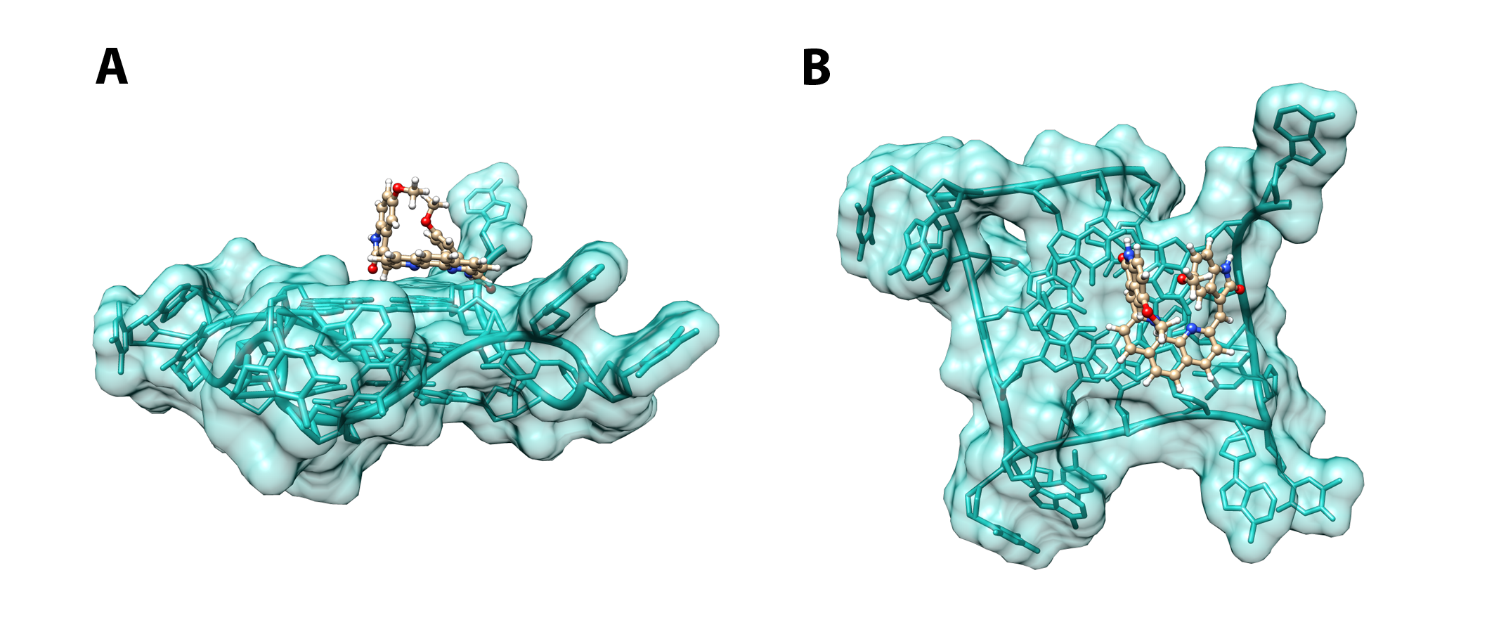


**Supplementary Figure 3. Binding pose of 2a on tel23-p.** Side (A) and top-view (B) of the binding pose of **2a** on **tel23-p** obtained by docking calculations.
